# Alkaline intracellular pH (pHi) activates AMPK–mTORC2 signaling to promote cell survival during growth factor limitation

**DOI:** 10.1016/j.jbc.2021.101100

**Published:** 2021-08-19

**Authors:** D. Kazyken, S.I. Lentz, D.C. Fingar

**Affiliations:** 1 Department of Cell and Developmental Biology, University of Michigan Medical School, Ann Arbor, Michigan, USA; 2 Division of Metabolism, Endocrinology and Diabetes, Department of Internal Medicine, University of Michigan Medical School, Ann Arbor, Michigan, USA

**Keywords:** AMPK, mTORC2, Akt, intracellular pH (pHi), AMPK, AMP-activated protein kinase, Rheb, Ras homolog enriched in brain, TSC, tuberous sclerosis complex, D-PBS, Dulbecco’s PBS, dFBS, dialyzed FBS, DKO, double KO, FBS, fetal bovine serum, HBSS, Hank’s balanced salt solution, MEFs, mouse embryonic fibroblasts, mTOR, mechanistic target of rapamycin, mTORC1, mTOR complex 1, mTORC2, mTOR complex 2, NH_4_Cl, ammonium chloride, pHi, intracellular pH

## Abstract

The mechanistic target of rapamycin (mTOR) complex 2 (mTORC2) signaling controls cell metabolism, promotes cell survival, and contributes to tumorigenesis, yet its upstream regulation remains poorly defined. Although considerable evidence supports the prevailing view that amino acids activate mTOR complex 1 but not mTORC2, several studies reported paradoxical activation of mTORC2 signaling by amino acids. We noted that after amino acid starvation of cells in culture, addition of an amino acid solution increased mTORC2 signaling. Interestingly, we found the pH of the amino acid solution to be alkaline, ∼pH 10. These observations led us to discover and demonstrate here that alkaline intracellular pH (pHi) represents a previously unknown activator of mTORC2. Using a fluorescent pH-sensitive dye (cSNARF1-AM) coupled with live-cell imaging, we demonstrate that culturing cells in media at an alkaline pH induces a rapid rise in the pHi, which increases mTORC2 catalytic activity and downstream signaling to the pro-growth and pro-survival kinase Akt. Alkaline pHi also activates AMPK, a canonical sensor of energetic stress. Functionally, alkaline pHi activates AMPK-mTOR signaling, which attenuates apoptosis caused by growth factor withdrawal. Collectively, these findings reveal that alkaline pHi increases mTORC2- and AMPK-mediated signaling to promote cell survival during conditions of growth factor limitation, analogous to the demonstrated ability of energetic stress to activate AMPK–mTORC2 and promote cell survival. As an elevated pHi represents an underappreciated hallmark of cancer cells, we propose that the alkaline pHi stress sensing by AMPK–mTORC2 may contribute to tumorigenesis by enabling cancer cells at the core of a growing tumor to evade apoptosis and survive.

The mechanistic target of rapamycin (mTOR) comprises the catalytic core of two distinct multiprotein complexes, mTOR complex 1 (mTORC1) and mTOR complex 2 (mTORC2). These mTORCs sense and integrate diverse extracellular and intracellular signals derived from hormones, growth factors, nutrients, and energy through distinct downstream substrates to control cell physiology in ways appropriate for biological context (1, 2, 3, 4, 5). mTORC1 primarily promotes anabolic cellular processes (*e.g.*, protein, lipid, and nucleotide synthesis) that sustain cell growth and cell proliferation, while mTORC2 controls cell metabolism, cell survival, and the actin cytoskeleton (1, 2, 3, 4, 5). Not surprisingly, altered mTORC1 and mTORC2 signaling contribute to pathologic conditions, including tumorigenesis and type II diabetes (4, 5).

Activation of mTORC1 by the cooperative action of insulin and amino acids has been studied extensively. In response to insulin, activation of the PI3K-Akt-tuberous sclerosis complex (TSC) pathway leads to Ras homolog enriched in brain (Rheb)-GTP–mediated activation of mTORC1 on the surface of lysosomes in a manner that requires sufficient levels of amino acids (4, 6, 7, 8). Amino acids load RagA/B proteins with GTP, which recruit mTORC1 to lysosomal membranes in proximity to Rheb (8, 9, 10, 11, 12, 13, 14). Through an induced proximity mechanism, Rheb-GTP in turn interacts with and activates mTORC1 through conformational changes (15, 16, 17). TSC and Rheb represent central signaling nodes at which growth factor and amino acid signals converge to effect mTORC1 regulation (4, 6, 7, 18). Insulin-PI3K-Akt signaling and amino acid sufficiency are required simultaneously to dissociate TSC from lysosomal membranes and away from Rheb, thus maintaining Rheb-GTP loading (19, 20, 21). Our knowledge of upstream pathways and mechanisms controlling mTORC2 activity and downstream signaling lags far behind that of mTORC1, however (4, 5). Activation of mTORC2 signaling by hormones and growth factors requires PI3K (4, 5, 22), and oncogenic Ras interacts with mTORC2 to increase its activity on the plasma membrane (23, 24). Curiously, nutrient withdrawal, specifically glutamine or glucose, activates mTORC2 (25, 26), and upregulation of the stress-sensing protein Sestrin2, which occurs during glutamine deprivation, increases mTORC2 activity (27, 28). In addition, our prior work demonstrated that during energetic stress, AMP-activated protein kinase (AMPK) directly activates mTORC2 to promote cell survival (26).

Considerable evidence indicates that amino acids are required for mTORC1 but not mTORC2 signaling (4, 5, 29, 30, 31, 32). Several studies reported paradoxical activation of mTORC2 signaling by amino acid stimulation, however (33, 34, 35, 36). The reason for this discrepancy in the literature remains unclear. While studying amino acid sensing by mTORC1, we noted that after amino acid starving cells in Dulbecco's PBS (D-PBS) or Dulbecco's modified Eagle's medium (DMEM), addition of a commercial amino acid solution but not re-feeding cells with complete DMEM (*i.e.*, containing amino acids) activated mTORC2 as well as AMPK. Interestingly, we measured the pH of the amino acid solution and found it to be an alkaline ∼pH 10. When we adjusted the pH of the amino acid solution to physiological pH 7.4, it failed to increase mTORC2 or AMPK signaling. These key observations enabled us to discover and demonstrate here that culturing cells in media at an alkaline pH activates the AMPK–mTORC2 axis through an increased intracellular pH (pHi), which attenuates apoptosis caused by growth factor withdrawal. As an elevated pHi represents an underappreciated hallmark of cancer cells (37, 38, 39), alkaline pH sensing by AMPK–mTORC2 may enable growth factor–deprived and nutrient-deprived cancer cells at the core of a growing tumor to evade apoptosis and survive.

## Results

### Amino acids at alkaline pH but not physiological pH increase mTORC2 and AMPK signaling

**Figure 1:**
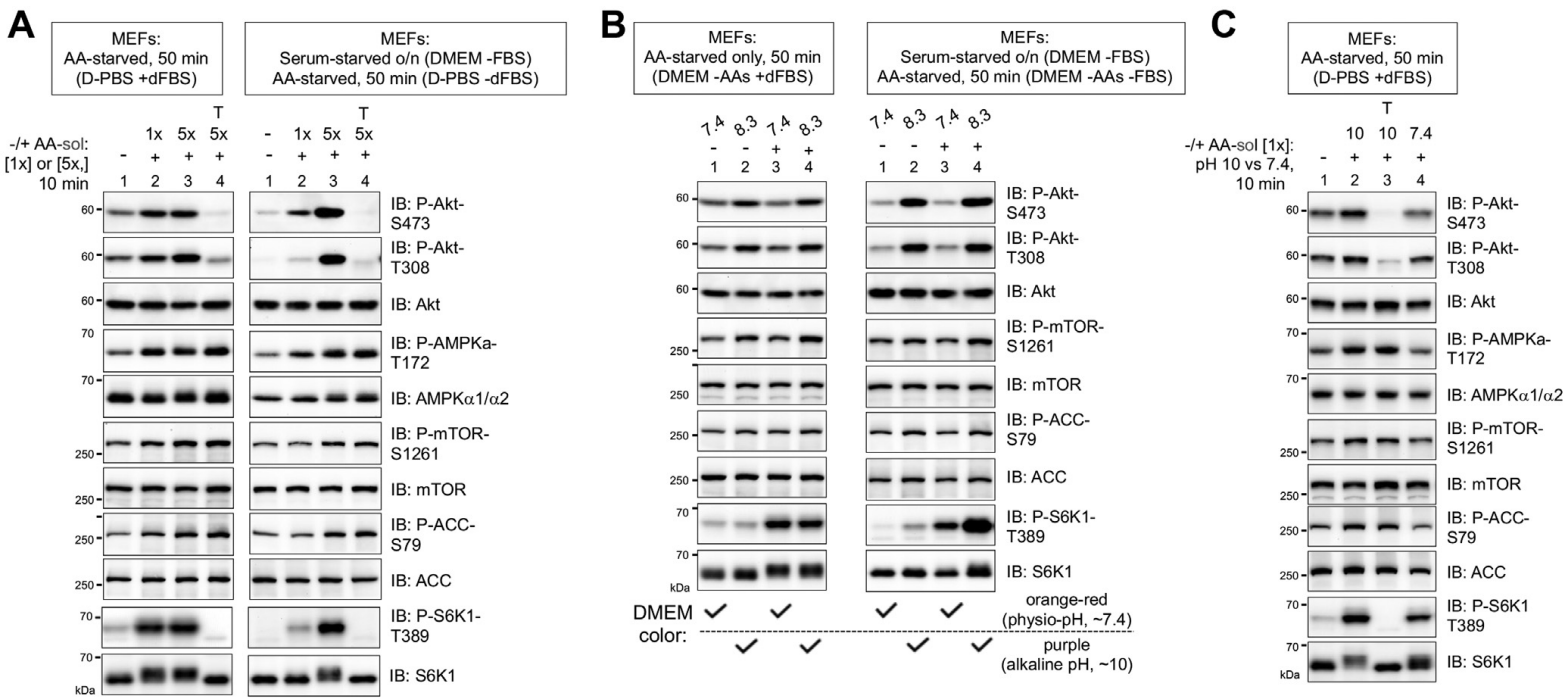
**Amino acid stimulation at alkaline but not physiological pH increases mTORC2 and AMPK signaling.***A*, MEFs were cultured in complete media (DMEM/FBS) (*left*) or serum-starved overnight, ∼16 h (*right*). They were next amino acid–starved in D-PBS (50 min) with (*left*) or without (*right*) dFBS, pretreated with Torin 1 (T) and treated without (−) or with (+) a commercial amino acid solution (amino acid–sol) (10 min) to [1×] or [5×] final. Whole-cell lysates were immunoblotted as indicated. Note that [1×]_f_ and [5×]_f_ represents roughly the amino acids found in RPMI or DMEM, respectively. *B*, MEFs were cultured as in panel *A* but amino acid–starved in amino acid–free DMEM and refed with DMEM at pH 7.3 or 8.3 lacking (−) or containing (+) amino acids (10 min). *C*, MEFs were amino acid–starved in D-PBS/dFBS, pre-treated with Torin 1 (T), and stimulated with an amino acid solution at pH 10 or 7.4 (10 min). AMPK, AMP-activated protein kinase; D-PBS, Dulbecco's PBS; dFBS, dialyzed FBS; MEFs, mouse embryonic fibroblasts; mTOR, mechanistic target of rapamycin; mTORC2, mTOR complex 2.

Researchers in the mTOR field use diverse methods to amino acid starve and stimulate cells. Shifting cells to media such as D-PBS, Hank's balanced salt solution (HBSS), or DMEM lacking amino acids is used to deprive cells of amino acids, whereas addition of an amino acid solution containing total (or individual) amino acids, or re-feeding of cells with complete media, is used to stimulate cells with amino acids. While studying the mTORC1 response to amino acids, we noted that after starving cells of amino acids in D-PBS (which contains glucose) with or without dialyzed fetal bovine serum (FBS) (dFBS), addition of a commercial amino acid solution increased mTORC2 signaling, as measured by the sensitivity of Akt S473 phosphorylation to the mTOR inhibitor Torin 1 (Fig. 1*A*, left and right panels). These results are consistent with a limited number of prior reports (33, 34, 35, 36). As mTORC2-mediated Akt S473 phosphorylation promotes and/or stabilizes Akt T308 phosphorylation in certain contexts (26, 40, 41), the amino acid solution also increased Torin 1–sensitive Akt T308 phosphorylation (Fig. 1*A*). Curiously, the amino acid solution increased phosphorylation of AMPKα on its activation loop site (S172) as well as the phosphorylation of mTOR S1261 and ACC S79, direct substrates of AMPK (Fig. 1*A*) (26, 42, 43). As expected, the amino acid solution increased S6K1 T389 phosphorylation in a Torin 1–sensitive manner, an established readout of mTORC1 signaling (Fig. 1*A*). On their face, these data agree with prior reports that amino acids increase mTORC2 signaling (33, 34, 35, 36) and AMPK signaling (34).

When we used DMEM lacking amino acids rather than D-PBS to amino acid deprive cells, however, addition of the amino acid solution induced a rapid change in DMEM color from orange-red to purple, indicating a change from physiological pH ∼7.4 to alkaline pH. Thus, we measured the pH of the amino acid solution and found it to be an alkaline pH ∼10. To compare how alkaline pH and amino acids control mTORC2 signaling, we adjusted the pH of DMEM lacking or containing amino acids to 7.4 or 8.3. DMEM at pH 8.3 but not 7.4 increased mTORC2 (*i.e.*, P-Akt-S473 and -T308) and AMPK (*i.e.*, P-mTOR-S1261; P-ACC-S79) signaling regardless of amino acid status in both the presence and absence of serum growth factors (Fig. 1*B*, left and right). As expected, DMEM containing but not lacking amino acids increased mTORC1 signaling (*i.e.*, S6K1 P-T389) regardless of the pH (Fig. 1*B*). Upon adjusting the pH of the amino acid solution, its addition at pH 10 but not 7.4 to cells in D-PBS (+dFBS) increased mTORC2 and AMPK signaling (Fig. 1*C*). These results indicate that the alkaline pH of the amino acid solution, not the amino acids *per se*, increased mTORC2 and AMPK signaling.

It is important to note that our findings confirm those of other laboratories that addition of a commercial amino acid solution to amino acid–starved cells increases mTORC2 as well as mTORC1 signaling (33, 34, 35, 36). Figure 1*A* (right) replicates the results of Tato *et al.* (33) using identical cell treatment conditions, while Fig. S1*A* replicates the results of Dalle Pezze *et al.* (34) in which addition of an amino acid solution (whose pH was not measured) to C2C12 myoblasts in HBSS increased mTORC2 signaling. When we controlled for the pH, however, addition of a pH 7.4 amino acid solution to C2C12 myoblasts starved in either HBSS (Fig. S1*A*) or DMEM (Fig. S1*B*) failed to increase mTORC2 signaling. Similar to our results, Dalle Pezze *et al.* (34) also reported that stimulation of starved cells with an amino acid solution activates AMPK. Collectively, these results provide strong evidence that increased pH not amino acids increases mTORC2 and AMPK signaling.

### Alkaline extracellular pH increases mTORC2 catalytic activity and signaling

**Figure 2:**
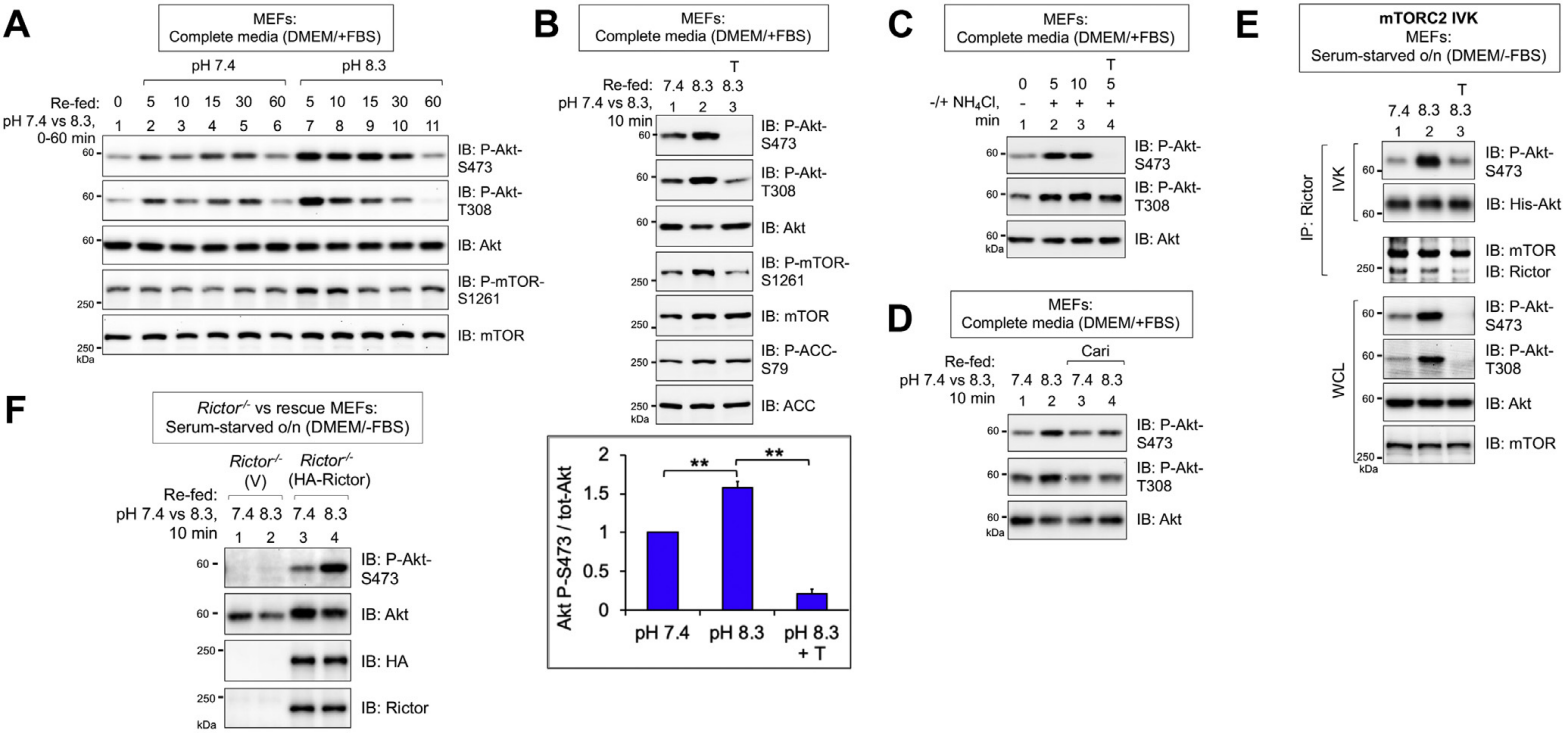
**Alkaline extracellular pH is sufficient to increase mTORC2 catalytic activity and signaling.***A*, MEFs in complete media (DMEM/FBS) were refed with the same media at either pH 7.4 or 8.3 for various times (5–60 min). Whole-cell lysates (WCLs) were immunoblotted as indicated. *B*, similar to panel *A*, except cells were pretreated with Torin 1 (T). Graph, mean ratio ± SD of Akt P-S473/Akt; n = 4 independent experiments. ∗∗*p* < 0.01 using one-way ANOVA and Tukey’s post hoc tests. *C*, MEFs in complete media (DMEM/FBS) were pretreated with Torin 1 (T) and stimulated without (−) or with (+) NH_4_Cl (5–10 min). *D*, similar to panel *A*, except cells were pretreated with cariporide (Cari) (30 min). *E*, Rictor was immunoprecipitated (IP) from MEFs that had been serum-starved, pretreated with Torin 1 (T), and refed with serum-free DMEM at pH 7.4 or 8.3 (10 min) −/+ Torin 1. *In vitro* kinase (IVK) reactions were performed with ATP and His-Akt1 substrate, with Torin 1 present in the IVK reaction (lane 3). IVKs and WCLs were immunoblotted as indicated. *F*, Rictor^−/−^ MEFs rescued with either vector control (V) or HA-Rictor were treated as in panel *A*. FBS, fetal bovine serum; MEFs, mouse embryonic fibroblasts; mTOR, mechanistic target of rapamycin; mTORC2, mTOR complex 2; NH_4_Cl, ammonium chloride.

We next tested whether culturing cells in media at an alkaline pH is sufficient to increase mTORC2 signaling in the absence of changes in amino acid levels. Re-feeding mouse embryonic fibroblasts (MEFs), HEK293T cells, and U2OS cells with complete DMEM (containing amino acids and FBS) at pH 8.3 but not pH 7.4 increased Akt phosphorylation (P-S473 and P-T308) (Fig. 2*A* and Fig. S2, *A*–*C*). These effects were rapid and transient, with maximal phosphorylation of Akt occurring at 5 to 15 min with apparent declines by 30 to 60 min. The activating effect of the alkaline extracellular pH on Akt phosphorylation was Torin 1 sensitive in all three cell lines (Fig. 2*B* and Fig. S2, *B* and *C*), thus confirming increased mTORC2 signaling. In MEFs, pH 8.3 to 8.5 mediated maximal mTORC2 signaling (Fig. S2*D*). These results demonstrate that the alkaline extracellular pH increases mTORC2 signaling, presumably by increasing the pHi. It is important to note that shifting cells to media at alkaline pH has been shown to increase the pHi rapidly (44, 45). To increase the pHi by an alternate method, we used ammonium chloride (NH_4_Cl). Addition of NH_4_Cl to cell culture media increases the cytosolic pH transiently upon rapid diffusion of NH_3_ (a weak base) into the cell and conversion to ammonium ion, NH_4_^+^, by scavenging protons from the cytosol, thus acidifying the cytosol (46, 47). Slower, subsequent entry of NH_4_^+^ into the cell attenuates the rise in pH upon dissociation into NH_3_ and H^+^. Note that NH_4_Cl is also used frequently to increase the pH of acidic organelles such as lysosomes (46). We found that treatment of MEFs with NH_4_Cl for 5 and 10 min increased mTORC2 signaling (Fig. 2*C*).

To confirm that culturing cells in media at alkaline pH activates mTORC2 by increasing the pHi, we used the drug cariporide. Cariporide inhibits NHE1, a H^+^–Na^+^ antiporter on the plasma membrane that drives proton (H^+^) efflux from the cytosol to the extracellular space. Thus, cariporide decreases the pHi (48, 49, 50). We therefore pretreated cells with cariporide before re-feeding with DMEM at alkaline pH to attenuate the rise in the pHi. Cariporide blunted the ability of DMEM at alkaline pH to increase mTORC2 signaling in MEFs, HEK293T cells, and U2OS cells (Fig. 2*D* and Fig. S2, *B* and *C*). Taken together, these results demonstrate that the increased pHi mediates the effect of the alkaline extracellular pH on mTORC2 signaling.

To test whether alkaline extracellular pH increases mTORC2 intrinsic catalytic activity, we performed mTORC2 *in vitro* kinase assays. To do so, we refed MEFs with DMEM, pH 8.3, immunopurified mTORC2 by immunoprecipitation of Rictor (a partner protein exclusive to mTORC2), and measured phosphorylation of His-Akt1 by mTORC2 *in vitro*. Indeed, the alkaline extracellular pH increased mTORC2 catalytic activity robustly, presumably by increasing the pHi (Fig. 2*E*). To demonstrate unambiguously that the increased pHi increases Akt S473 phosphorylation *via* mTORC2, we used Rictor^−/−^ MEFs. Indeed, refeeding with DMEM, pH 8.3, increased P-Akt-S473 in Rictor^−/−^ MEFs rescued with HA-Rictor but not vector control (V) (Fig. 2*F*). Taken together, these results show that a more alkaline pHi activates mTORC2 to increase downstream signaling to Akt.

### Incubation of cells in media at alkaline pH or containing NH_4_Cl increases pHi

**Figure 3:**
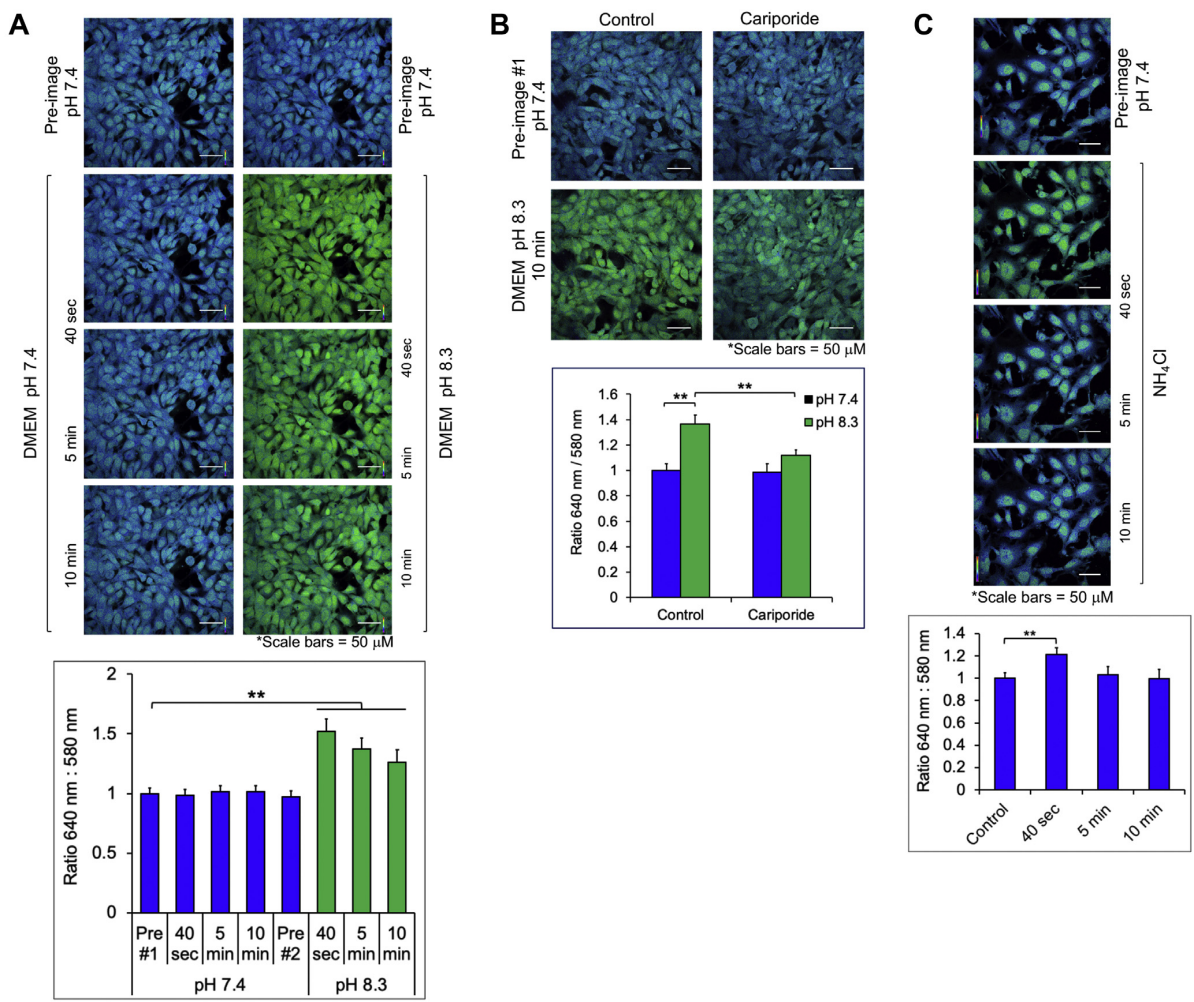
**Incubation of cells in media at alkaline pH or containing NH**_**4**_**Cl increases intracellular pH (pHi).***A*, MEFs were preloaded with cSNARF-1-AM in serum-free DMEM, pH 7.4 (30 min). They were then refed with DMEM/FBS, pH 7.4, and one image set was acquired (preimage #1 pH 7.4). The cells were refed again with the same media, and three image sets were acquired at 40 s, 5 min, and 10 min. At this point, the cells were refed once more with DMEM/FBS at pH 7.4, and another image set was acquired (preimage #2 pH 7.4). Next, the cells were refed with DMEM/FBS at pH 8.3, and three image sets were acquired at 40 s, 5 min, and 10 min. Ratiometric (640:580 nm) pseudocolored images are shown for each treatment condition. *Green images* reflect the increased pHi (*i.e.*, less-protonated cSNARF1-AM). Graph, quantitation of ratiometric images; n = 100 cells from four fields (∼25 cells/field) ± SD. ∗∗*p* < 0.01 using one-way ANOVA and Tukey’s post hoc tests. *B*, MEFs were preloaded with cSNARF-1-AM and treated and analyzed as above, except that cariporide was included during cSNARF1-AM loading (30 min). The cells were then washed once in serum-free media and refed with DMEM/FBS at pH 7.4 or 8.3 without (control) or with cariporide (10 min). Graph, quantitation of ratiometric images as above in panel *A*. (n = 100 cells). *C*, MEFs were loaded with cSNARF-1-AM and treated and analyzed as in panel *A*, except image sets were acquired from cells incubated in DMEM/FBS, pH 7.4, without (preimage) or with NH_4_Cl for 40 s, 5 min, or 10 min. Ratiometric (640:580 nm) pseudocolored images are shown for each treatment condition. Graph, quantitation of ratiometric images as above in panel *A* (n = 100 cells). FBS, fetal bovine serum; MEFs, mouse embryonic fibroblasts; NH_4_Cl, ammonium chloride.

We next confirmed that incubation of cells in media at alkaline pH increases the pHi. To do so, we used live-cell imaging coupled with a cell-permeable, pH-sensitive fluorescent dye (cSNARF1-AM) that is capable of measuring changes in the pHi between 7 and 8. cSNARF1-AM undergoes a pH-sensitive shift in fluorescence wavelength emission depending on the protonation state. With 488-nm excitation, peak emission occurs at 580 nm in the protonated state (more acidic) and 640 nm in the deprotonated state (more alkaline). Thus, ratiometric imaging (640:580 nm) enables detection of changes in the pHi within the physiological range of normal cells (∼pH 7.2) and cancer cells (∼pH 7.4–7.6) (38, 51). MEFs were preloaded with cSNARF1-AM and then refed with complete DMEM at pH 7.4 for 10 min followed by refeeding with media at pH 8.3 for 10 min. Visualization of the acquired pseudocolored ratiometric images (640:580 nm) and quantitation of the signal ratios revealed a rapid and significant increase in the pHi when cells were shifted to DMEM at alkaline pH (green cells) relative to those maintained in media at physiological pH 7.4 (blue cells) (Fig. 3*A*). To confirm that cariporide attenuates the increase in the pHi caused by shifting cells to media at alkaline pH, we pretreated cells with cariporide before the shift to DMEM, pH 8.3, and monitored the pHi using cSNARF1-AM and live-cell imaging. It is important to note that the NHE family consists of several family members, and cariporide inhibits only a subset with differing potencies; in addition, cells express other proton (H+) transporters (49). Thus, cariporide would be expected to attenuate increases in the pHi modestly. We found that, indeed, cariporide partially blocked the increase in the pHi (Fig. 3*B*). In addition, we confirmed that addition of NH_4_Cl to DMEM at physiological pH 7.4 induces a rapid but transient rise in the pHi, as expected (Fig. 3*C*). These results confirm that shifting cells to media at alkaline pH or addition of NH_4_Cl to media at the physiological pH leads to a rapid increase in the pHi, consistent with other studies (44, 45).

### AMPK promotes mTORC2 signaling and cell survival in response to alkaline pHi

**Figure 4:**
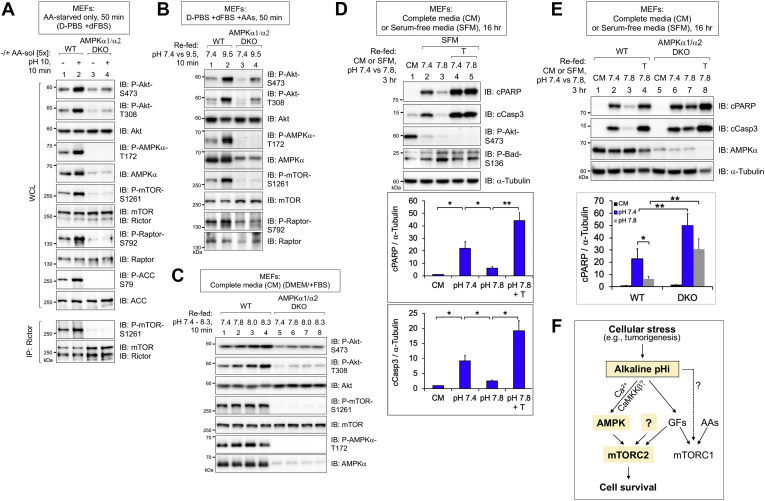
**AMPK promotes mTORC2 signaling and suppresses apoptosis in response to alkaline extracellular pH.***A*, WT and AMPKα1/α2 double KO (DKO) MEFs were amino acid–starved in D-PBS +dFBS (50 min) and stimulated with an amino acid solution (amino acid–sol) whose pH had not been adjusted (*i.e.*, pH = 10) to [5×] final (10 min). Rictor was immunoprecipitated (IP), and whole-cell lysates (WCLs) and immunoprecipitates were immunoblotted as indicated. *B*, WT and AMPK DKO MEFs were cultured in D-PBS +dFBS containing 1× amino acids and refed with the same media at pH 7.4 or 9.5 (10 min). Note that pH 9.5 is the pH of D-PBS +dFBS supplemented with pH 10 amino acid to 1× final. *C*, WT and AMPK DKO MEFs in complete media (DMEM/FBS) were refed with the same media at various pH values, 7.4 to 8.3 (10 min). *D*, WT MEFs cultured in complete media (CM) (DMEM/FBS) or serum-free media (SFM) (DMEM/-FBS) for 16 h were refed with CM at pH 7.4 or SFM at pH 7.4 or 7.8 without or with Torin 1 (T) for an additional 3 h (19 h total). Graph, mean ratio ± SD of cParp/tubulin and cCasp3/tubulin. n = 4 experiments. ∗*p* < 0.05, ∗∗ *p* < 0.01 using one-way ANOVA and Tukey’s post hoc tests. *E*, WT and AMPKα1/α2 DKO MEFs were treated as in panel *D*. Graph, mean ratio ±SD of cParp/tubulin. n = 5 experiments. ∗*p* < 0.05, ∗∗ *p* < 0.01 using ANOVA as above. *F*, model. Alkaline pHi activates AMPK-mTORC2 signaling, which attenuates apoptosis caused by growth factor withdrawal. AMPK, AMP-activated protein kinase; cParp, cleaved Parp; D-PBS, Dulbecco's PBS; dFBS, dialyzed FBS; MEFs, mouse embryonic fibroblasts; mTOR, mechanistic target of rapamycin; mTORC2, mTOR complex 2.

Our prior work demonstrated that energetic stress increases mTORC2 catalytic activity and signaling directly through AMPK-mediated phosphorylation of mTOR and mTORC2 partner proteins (*e.g.*, Rictor) (26). As the AMPK–mTORC2 axis responds to energetic stress, we speculated that it may also respond to alkaline pH stress. We therefore compared the ability of the alkaline pHi to increase mTORC2 signaling in WT and AMPKα1/α2 double KO MEFs (*i.e.*, AMPK DKO). When MEFs incubated in D-PBS/dFBS were stimulated with the alkaline amino acid solution or refed with D-PBS/dFBS adjusted to pH 9.5 (the pH of D-PBS/dFBS after addition of the amino acid solution to 1× final), we found that alkaline extracellular pH increased mTORC2 signaling in a manner partly dependent on AMPK (Fig. 4, *A* and *B*). As expected, AMPK DKO abrogated AMPK signaling (P-AMPK-S172; P-mTOR-S1261; P-Raptor-S792) (Fig. 4, *A* and *B*). Alkaline extracellular pH was also sufficient to increase mTORC2 signaling in a manner partly dependent on AMPK in cells cultured in complete media (*i.e.*, DMEM/FBS) (Fig. 4*C*). From these results, we conclude that alkaline pHi increases mTORC2 signaling through AMPK and another unknown signal(s).

We next investigated how alkaline pHi activates AMPK. As the increased pHi has been reported to mobilize Ca^2+^ stores and raise cytosolic Ca^2+^ (52, 53), and as the Ca^2+^-dependent, noncanonical kinase CaMKKβ phosphorylates and activates AMPKα, we asked whether Ca^2+^ and CaMKKβ are required for the alkaline extracellular pH to increase mTORC2 signaling. Note that two distinct upstream kinases, LKB1 and CaMKKβ, phosphorylate AMPKα on its activation loop site (S172) to activate AMPK depending on context (42, 54). We therefore used MEFs lacking LKB1 to study the sole role of CaMKKβ in AMPK activation. We found that pretreatment of LKB1^−/−^ MEFs with STO-609, a small molecular inhibitor of CaMKKβ, or Bapta-AM, a Ca^2+^ chelator, blunted or ablated, respectively, the ability of DMEM, pH 8.3, to increase mTORC2 signaling to Akt (Fig. S4, *A* and *B*). As expected, STO-609 reduced the ability of DMEM, pH 8.3, to activate AMPK, as shown by reduced P-AMPK-S172 and P-mTOR-S1261 (Fig. S4*A*). These results suggest that Ca^2+^ and CaMKKβ are required for activation of AMPK and mTORC2 signaling in response to the increased pHi.

Cell survival requires sufficient levels of growth factors, with Akt functioning as an important survival kinase (55). Our recent work demonstrated that AMPK–mTORC2 signaling promotes cell survival in response to acute energetic stress (26). We therefore tested the hypothesis that an elevated pHi protects against apoptosis during growth factor limitation, a setting common for growing tumors. We therefore serum-starved MEFs overnight (16 h) in DMEM and then refed the cells with either complete DMEM (*i.e.*, with FBS) or serum-free DMEM (*i.e.*, without FBS) for an additional 3 h. As expected, cells maintained in serum-free media for the full 19 h displayed increased apoptosis relative to those rescued with complete media for the last 3 h, as monitored by blotting for cleaved caspase 3 and cleaved Parp (Fig. 4*D*). Consistent with our hypothesis that alkaline pHi protects against apoptosis, refeeding MEFs with serum-free DMEM at alkaline pH 7.8 (whose pH had been adjusted using sodium bicarbonate rather than NaOH, which maintains DMEM pH for a longer period of time) suppressed apoptosis (Fig. 4*D*). Importantly, Torin 1 blocked the ability of DMEM at alkaline pH to suppress apoptosis, indicating a requirement for mTOR activity. While the prolonged alkaline extracellular pH (*i.e.*, 3 h) could not maintain Akt S473 phosphorylation, it maintained inhibitory phosphorylation of the Akt substrate and proapoptotic protein BAD (P-S136) (Fig. 4*D*). Consistent with our hypothesis that AMPK is required for alkaline pHi to protect against apoptosis, DKO of AMPKα1/α2 partially suppressed the ability of serum-free DMEM at alkaline pH 7.8 to attenuate apoptosis caused by growth factor withdrawal (Fig. 4*E* and Fig. S3).

## Discussion

This study identifies the alkaline pHi as a previously unrecognized activator of AMPK and mTORC2 that attenuates apoptosis during growth factor limitation (Fig. 4*F*). It is important to note that a recent study also found that the alkaline pHi increases Akt P-S473, although mechanistic details were not defined (44). As AMPK promotes mTORC2 signaling and cell survival mediated by the alkaline pHi in part rather than in full, other signals likely cooperate with AMPK to activate mTORC2 in response to the alkaline pHi. In support of this idea, the alkaline pHi and amino acids produced an additive effect on mTORC1 signaling to S6K1 (Fig. 1*B*-right), suggesting that another unknown signal responds to the alkaline pHi to increase mTORC1 and perhaps mTORC2 signaling (Fig. 4*F*). We speculate that prior studies reporting activation of mTORC2 signaling by amino acids (33, 34, 35, 36) likely mistook an increase in the pHi for an increase in amino acid levels. In fact, Tato *et al.* (33). found that amino acids selectively activate mTORC2 signaling depending on the method of amino acid starvation and stimulation used: addition of a commercial amino acid solution to starved cells but not refeeding with complete DMEM increased mTORC2 signaling. By controlling for the pH of the amino acid solution or using DMEM, pH 7.4, lacking or containing amino acids, our results reveal that alkaline extracellular pH (and thus alkaline pHi), not amino acids, increases mTORC2 signaling, thus correcting a misconception in the mTOR literature. Increased pHi also likely accounts for the activation of AMPK caused by addition of an amino acid solution to cells, as observed by Dalle Pezze *et al.* (34).

Our findings suggest a mechanism for how alkaline pHi activates AMPK–mTORC2 signaling. CaMKKβ inhibition or Ca^2+^ chelation blunted the ability of media at alkaline pH to increase AMPK activity and mTORC2 signaling (see Fig. S4, *A* and *B*). We thus propose that alkaline pHi increases cytosolic Ca^2+^ and activates CaMKKβ, which results in CaMKKβ-mediated activation of AMPK (42, 43) and AMPK-mediated activation of mTORC2 (26). In support of this idea, increased pHi was reported to mobilize Ca^2+^ stores (52, 53). Additional studies will be required to test this model. Interestingly, Merhi *et al.* (56) reported that the metabolic waste product ammonium (NH_4_^+^), delivered to cells as either NH_4_Cl or NH_4_OH, activates mTORC2 signaling in a manner that requires Ca^2+^ mobilization. Although they did not determine the mechanism by which NH_4_Cl or NH_4_Cl-induced Ca^2+^ mobilization increases mTORC2 signaling, they speculated involvement of a pH-mediated effect. Our results may explain their results, at least in part.

Dysregulated pH represents an underappreciated hallmark of the cancer cell microenvironment, with cancer cells displaying a reversal in the pH gradient mediated by altered proton (H^+^) flux (*i.e.*, elevated pHi and decreased extracellular pHe, the reverse of normal cells) (37, 38, 39, 49, 50, 51). While traditional thought presumed that cancer cells possess an acidic cytosol due to reliance on aerobic glycolysis (*i.e.*, the Warburg effect) and production of metabolic acids, many cancer cells in fact possess a slightly basic cytosol (37, 38, 51). Functionally, dysregulated pH dynamics modifies cancer cell behaviors, with increased pHi driving cell proliferation, survival, migration, and metastasis. Changes in the pHi control the structure and function of pH-sensitive proteins (aka, pH sensors) through protonation/deprotonation, a post-translational modification akin to phosphorylation, ubiquitination, and so forth (37, 38, 39, 51). Documented pH sensors with recurring charge-changing mutations (*e.g.*, Arg to His) include p53, EGF receptor, Ras-GRP1, and β-catenin (47, 57, 58, 59). Elevated activity of several plasma membrane ion exchangers, including the Na^+^–H^+^ exchanger NHE1, contributes to the increased pHi and correlates with tumor initiation, progression, and metastasis (38, 49). Indeed, increased H^+^ efflux contributes to transformed cell behaviors mediated by oncogenic Ras, as demonstrated in human mammary cells and a *Drosophila* model (60).

By identifying the alkaline pHi as an activator of AMPK–mTORC2 signaling that attenuates apoptosis caused by growth factor withdrawal, our work suggests that the AMPK–mTORC2 axis may drive tumorigenesis in response to dysregulated pH dynamics by enabling growth factor–deprived, nutrient-deprived, and oxygen-deprived cancer cells at the core of a growing tumor to survive. Such a role is consistent with the paradoxical role of AMPK as a tumor promoter in certain contexts (despite its established role as a tumor suppressor) and the newfound role for AMPK–mTORC2 signaling in cell survival during energetic stress (26, 61, 62, 63, 64, 65, 66). More broadly, our results suggest that the AMPK–mTORC2 axis senses diverse types of cellular stress, which likely rewires cell metabolism to help cells adapt and survive. In the future, it will be important to identify the pH sensors that transduce signals to AMPK and mTORC2.

## Experimental procedures

### Cell culture

Cell lines (MEFs; HEK293T; C2C12; U2OS) were cultured in DMEM containing high glucose [4.5 g/l], glutamine [584 mg/l], and sodium pyruvate [110 mg/l] (Life Technologies #11995-065) supplemented with 10% FBS (Life Technologies #10347-028) and incubated at 37 °C in a humidified atmosphere with 7.5% CO_2_. Rictor^−/−^ MEFs rescued with vector control or HA-Rictor were generated as described (26). To effect amino acid starvation, cells were cultured in either D-PBS (which contains [1 g/l] D-glucose) (Life Technologies #14287-080) or amino acid–free DMEM (United States Biological #D9800-13) without or with 10% dFBS (Life Technologies #A33820-01) for 50 min. To effect serum starvation, cells were cultured in DMEM containing 20 mM Hepes, pH 7.2, for ∼16 h overnight. Cells were amino acid –stimulated in two ways: (1) An amino acid solution (RPMI 1640 Amino Acid Solution [50×]) (Sigma # R7131) supplemented with L-glutamine (Sigma # G8540) was added to cells in amino acid–free media to a final concentration of 1× (∼concentration in RPMI) or 5× (∼concentration in DMEM). The pH of the amino acid solution was either not adjusted (∼pH 10) or adjusted to pH 7.4 with 1 M HCl or (2) cells were refed with amino acid–replete DMEM after incubation in amino acid–free DMEM. To treat cells at various alkaline extracellular pHs acutely, the pH of D-PBS or DMEM was adjusted with NaOH. To stably maintain DMEM at an alkaline pH (*i.e.*, pH 7.8), the NaHCO_3_ concentration was increased from 44 mM to 120 mM by directly dissolving in NaHCO_3_ followed by filter sterilization and equilibration overnight in an incubator at 7.5% CO_2_, as described (45). As an alternate method to increase pHi, NH_4_Cl [10 mM] was added to the cell culture media.

### Live-cell imaging of cSNARF1-AM

MEFs were plated in 8-well cover glass bottom chambers (Lab-tek #155409) and loaded with cSNARF-1-AM [10 μM] (Thermo Fisher #C1272), a fluorescent pH-sensitive dye, for 30 min at 37 °C in serum-free DMEM, which facilitates cSNARF1-AM loading. The cells were then rinsed with serum-free DMEM and refed with DMEM/FBS [10%], pH 7.4. Live-cell imaging (0–10 min) of cells cultured in DMEM/FBS, pH 7.4 or 8.3, was performed using a Nikon A1 confocal microscope equipped with a stage-top incubator that maintains the temperature and CO_2_. Images were acquired using a 40× oil objective (1.3 NA) at a resolution of 1024 × 1024 and an optical thickness of 1.18 μm (confocal aperture set at 3 airy units). cSNARF1-AM signal was excited with an argon laser at 488 nm, and images sets were collected simultaneously in two emission band-pass filters at 553 to 618 nm (for 580 nm) and 663 to 738 nm (for 640 nm). Nikon NIS-Elements software was used to create and pseudocolor the 640:580 nm ratiometric images with values ranging from 0 (violet) to 2.0 (red). MetaMorph software was used to quantify and graph the ratiometric images by measuring the average ratio of a region of interest within one cell. Twenty five cells per field times four fields were quantified (n = 100 cells). A change in the 640:580 nm ratio and accompanying pseudocolored image reflects a change in the pHi.

## Data availability

All data are contained in this article.

## Supporting information

This article contains supporting information.

## Conflict of interest

The authors declare that they have no conflicts of interest with the contents of this article.

## Supporting information

Supplemental Experimental Procedures and Figures S1–S4
