# Supplementary material for: Alkaline intracellular pH (pHi) activates AMPK–mTORC2 signaling to promote cell survival during growth factor limitation
Source: J Biol Chem. 2021 Aug 19;297(4):101100. doi: 10.1016/j.jbc.2021.101100 (PMC8479482; doi:10.1016/j.jbc.2021.101100)

Supporting Information

Kazyken *et al.*

Alkaline intracellular pH (pHi) activates AMPK-mTORC2 signaling to promote cell survival during growth factor limitation

Supplemental Experimental Procedures

Materials

General chemicals were from Thermo Fisher Scientific or Sigma Aldrich. NP40, Brij35, and CHAPS detergents were from Pierce; cOmplete Protease Inhibitor Cocktail (EDTA-free) tablets were from Millipore Sigma (#11836170001); protein A-Sepharose CL-4B was from Sigma-Aldrich (#17-0780-01); Immobilon-P polyvinylidene difluoride (PVDF) membrane (0.45 μM) was from Millipore; reagents for enhanced chemiluminescence (ECL) were from Alkali Scientific (Bright Star #XR92) or Advansta (WesternBright Sirius HRP substrate); NH_4Cl was from Fisher (#A661); ST0-609 was from Cayman (#1532510); PageRuler (#26617) and PageRuler Plus (#26619) prestained protein ladders, as well as Bapta-AM (#B1205), were from Thermo Fisher.

Antibodies

The following antibodies were from Cell Signaling Technology (CST): AMPKα P-T172 (#4188); pan-AMPKα (#2532); Akt P-S473 (#4060); Akt P-T308 (#4056); Akt (#9272); mTOR (#2972); raptor P-S792 (#2083); raptor (#2280); S6K1 P-T389 (#9234); ACC P-S79 (#3661); ACC (#3676); Bad P-S136 (#4366); cleaved caspase 3 (#9664); cleaved PARP (#9544); α-tubulin (#2144); mTOR P-S2481 was from Millipore (#09-343). The following custom polyclonal anti-peptide antibodies were generated by us with the aid of Covance, as described previously (1): mTOR P- S1261 (amino acids 1256-1266; rat); Rictor (amino acids 6-20; human); and S6K1 (amino acids 485-502; rat 70 kDa isoform). Donkey anti-rabbit-HRP secondary antibody was from Jackson (#711-095-152).

Drug treatments, cell lysis, immunoprecipitation, and western blotting

Cells were pre-treated with Torin1 [100 nM] (30 min.) (shared by D. Sabatini, MIT and Whitehead Institute, Boston, MA) or cariporide [10 μM] (30 min.) (Sigma #SML1360) prior to lysis. Unless otherwise indicated, cells were washed 2x in PBS and lysed in buffer containing NP-40 [0.5%] and Brij35 [0.1%], as described (1). Lysates were incubated on ice (15 min.) and then spun at 13,200 rpm (5 min.) at 4°C. Post-nuclear supernatants were normalized for protein levels by Bradford assay. For immunoprecipitation, whole cell lysates were incubated with antibodies for 2 hr. at 4°C and Protein A-Sepharose beads for 1 hr. Beads were washed three times in lysis buffer and resuspended in 1x sample buffer. Samples were resolved on SDS-PAGE and transferred to PVDF membranes in Towbin transfer buffer containing 0.02% SDS. Western blotting was performed by blocking PVDF membranes in Tris-buffered saline (TBS) pH 7.5 with 0.1% Tween-20 (TBST) containing 3% non-fat dry milk, as described, and incubating the membranes in TBST/ BSA (2%) containing primary or secondary HRP antibodies. Blots were developed by ECL and detected digitally with a Chemi-Doc-It System (UVP).

mTORC2 *in vitro* kinase assays

mTORC2 in vitro kinase (IVK) assays were performed, as described (2,3). Briefly, serum starved MEFs were re-fed with DMEM pH 7.4 or pH 8.3 and lysed in buffer containing CHAPS [0.3%]. Rictor was immunoprecipitated from a near confluent 10 cm plate and incubated with ATP [250 μM] and recombinant His-Akt1 [100 ng/reaction] (Millipore #14-279) in 15 μl kinase buffer (25 mM HEPES; 100 mM potassium acetate; 1 mM $MgCl_2$) at 30°C for 30 min. and stopped by addition of sample buffer followed by incubation at 95°C for 10 min.

Image editing

Adobe Photoshop was used for preparation of western blot images, using only levels, brightness, and/or contrast equivalently over the entire image. All presented images reflect the raw images.

Statistical analysis
Fiji was used to quantitate ECL western blot signals. Results are presented as mean ± SD. Significance of the difference between two measurements was determined by Student's t test. Multiple comparisons were analyzed for significance using one-way ANOVA followed by pairwise Tukey's post hoc tests. Values of $p < 0.05$ were considered significant. All experiments were performed at least three times, if not more, unless indicated otherwise in the figure legend.

Supplementary Figure Legends

Supplementary Figure S1: Activation of mTORC2 signaling by alkaline extracellular pH in C2C12 myoblasts (related to figure 1)
S1A. C2C12 cells were serum and amino acid starved overnight (~16 hrs.) in HBSS and stimulated with an amino acid solution at pH 7.4 or pH 10 to 1x final (10 min.). Whole cell lysates were immunoblotted as indicated.
S1B. C2C12 cells were serum starved overnight in DMEM (~16 hrs.), shifted to amino acid-free DMEM lacking FBS (50 min), and then stimulated with an amino acid solution at pH 7.4 or pH 10 to 1x final (10 min).

Supplementary Figure S2: Alkaline extracellular pH increases mTORC2 signaling in HEK293T and U2OS cells (related to figure 2)
S2A. HEK293T cells were re-fed with complete media (DMEM/FBS) (30 min) and then re-fed again with the same media at pH 7.4 or 8.3 for various times (0-60 min). Whole cell lysates were immunoblotted as indicated.
S2B. HEK293T cells were pre-treated without or with Torin1 (T) or cariporide (C) (30 min.) and re-fed with complete media (DMEM/FBS) at pH 7.4 or 8.3 (15 min) without or with drugs.
S2C. U2OS cells were treated as in **C.**
S2D. MEFs in complete media (DMEM/FBS) were re-fed with media at various pH values (pH 7.4-9.0) (10 min).

Supplementary Figure S3. AMPK suppresses apoptosis in response to alkaline extracellular pH: WT and AMPKα1/α2 DKO MEFs cultured in complete media (CM) (DMEM/+FBS) or serum-free media (SFM) (DMEM/-FBS) for 16 hrs. were re-fed with CM at pH 7.4 or SFM at pH 7.4 or 7.8 without or with Torin1 (T) for an additional 3 hrs. (19 hrs. total). Graph, mean ratio $\pm$ SD of cCasp3/ tubulin. n = 4 experiments. *$p < 0.05$, n.s., not significant, using one-way ANOVA and Tukey's post hoc tests.

Supplementary Figure S4. CaMKKβ inhibition or Ca^{2+} chelation reduces mTORC2 signaling in response to alkaline extracellular pH:
S4A. LKB1$^{-/-}$ MEFs in complete media (DMEM/+FBS) were pre-treated without (DMSO) or with ST0-609 (30 min.) and re-fed with the same media at pH 7.4 or 8.3 (10 min.) lacking or containing ST0-609. Whole cell lysates were immunoblotted as indicated.
S4B. Similar to S4**A**, except cells were pre-treated and re-fed with complete media lacking (DMSO) or containing Bapta-AM (T).

Supplementary References

1. Acosta-Jaquez, H. A., Keller, J. A., Foster, K. G., Ekim, B., Soliman, G. A., Feener, E. P., Ballif, B. A., and Fingar, D. C. (2009) Site-specific mTOR phosphorylation promotes mTORC1-mediated signaling and cell growth. *Mol Cell Biol* **29**, 4308-4324
2. Huang, J., Dibble, C. C., Matsuzaki, M., and Manning, B. D. (2008) The TSC1-TSC2 complex is required for proper activation of mTOR complex 2. *Mol Cell Biol* **28**, 4104-4115

3. Kazyken, D., Magnuson, B., Bodur, C., Acosta-Jaquez, H. A., Zhang, D., Tong, X., Barnes, T. M., Steinl, G. K., Patterson, N. E., Altheim, C. H., Sharma, N., Inoki, K., Cartee, G. D., Bridges, D., Yin, L., Riddle, S. M., and Fingar, D. C. (2019) AMPK directly activates mTORC2 to promote cell survival during acute energetic stress. *Sci Signal* **12**

Supplementary Figure S1
(related to Figure 1)

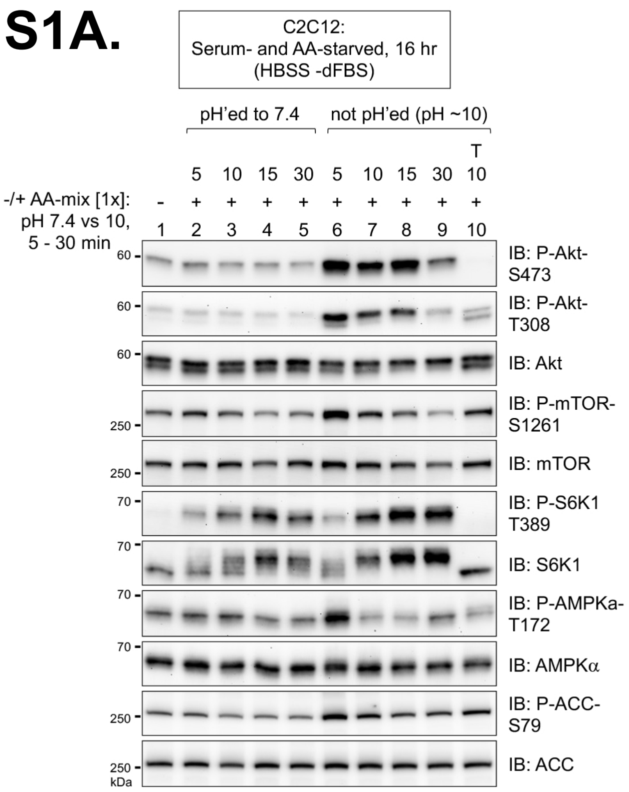

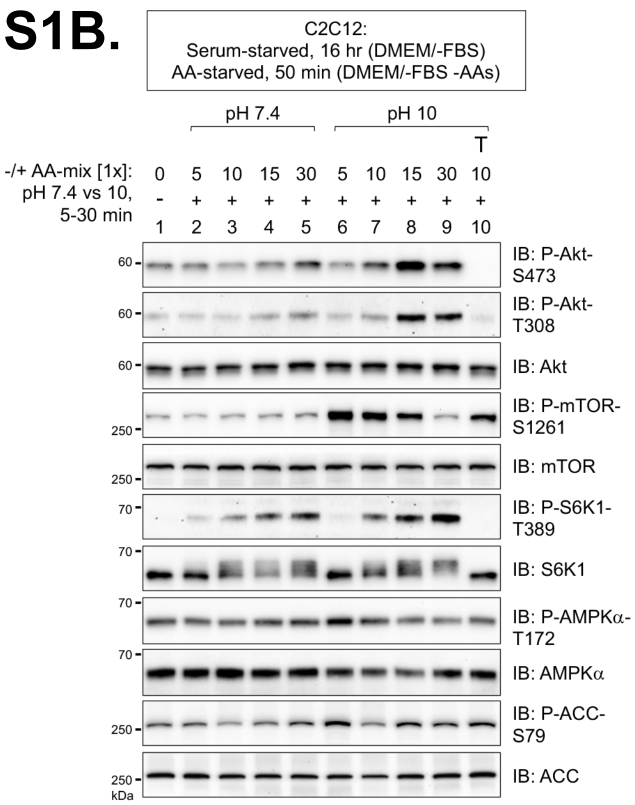

Supplementary Figure S2
(related to Figure 2)

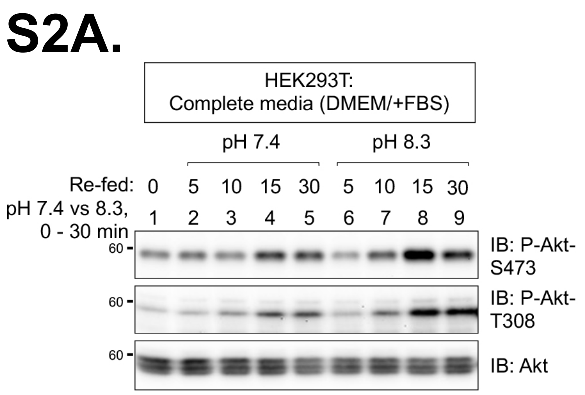

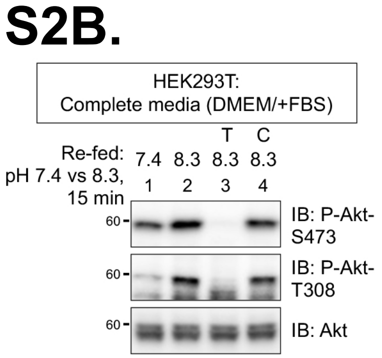

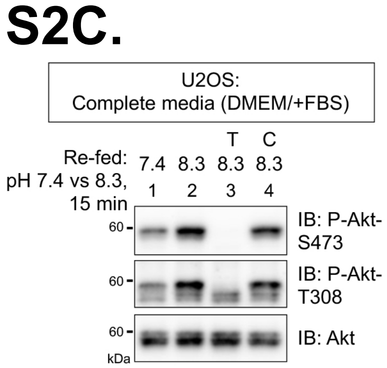

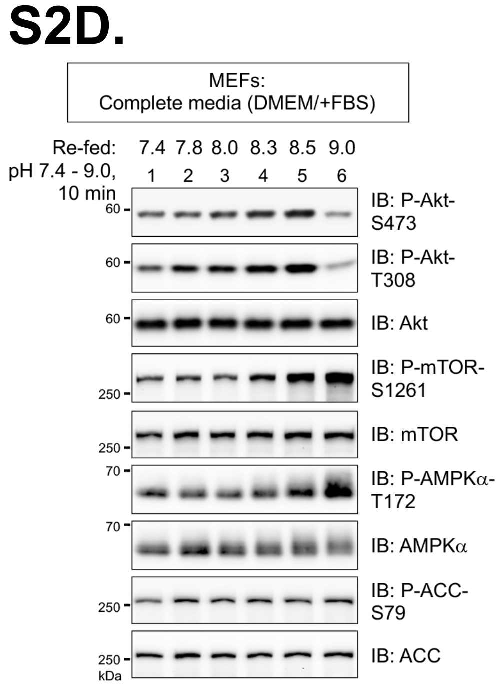

Supplementary Figure S3
(related to Figure 4)

S3.

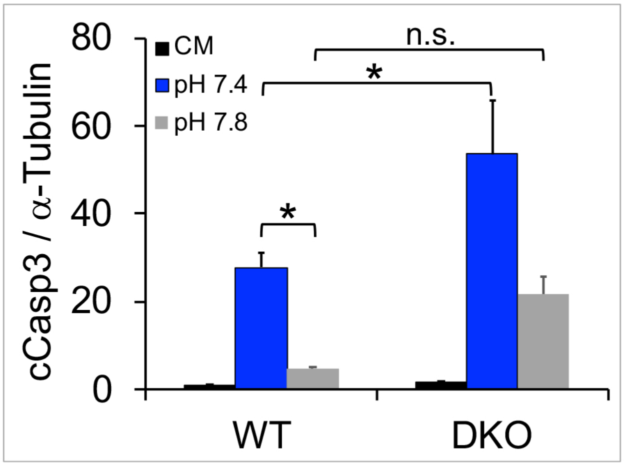

Supplementary Figure S4
(related to Figure 4)

S4A.

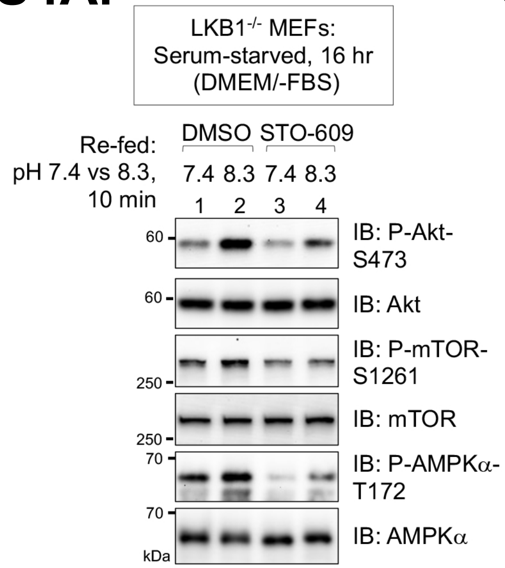

S4B.

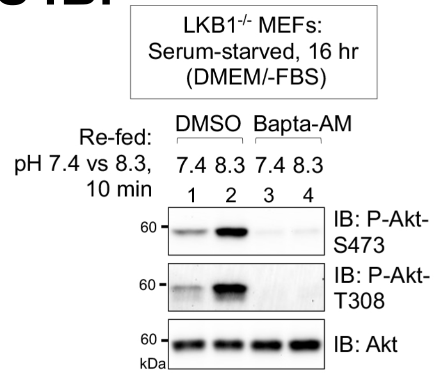

Supplement: Supplemental Experimental Procedures and Figures S1–S4 [file mmc1.pdf]
